# Evaluating the progression to abnormal thyrotropin in euthyroid preconception women: a population-based study

**DOI:** 10.1186/s13044-024-00192-w

**Published:** 2024-03-11

**Authors:** Rili Gao, Xinyi Lyu, Ying Yang, Jinrong Fu, Chuanyu Zhao, Haixia Guan, Xu Ma

**Affiliations:** 1 Department of Endocrinology, Guangdong Provincial People’s Hospital (Guangdong Academy of Medical Sciences), Southern Medical University, Guangzhou, China; 2 National Research Institute for Family Planning, Beijing, China; 3 National Human Genetic Resources Center, Beijing, China; 4 Graduate School of Peking Union Medical College, Beijing, China

**Keywords:** Thyrotropin, Thyroid dysfunction, Thyrotropin abnormalities, Population-based study

## Abstract

**Background:**

Abnormal preconception thyrotropin levels were associated with fecundability and adverse fetomaternal outcomes, however, little is known regarding the natural change of serum thyrotropin in euthyroid preconception women. Thus, we performed a population-based study to evaluate the progression to abnormal thyrotropin in euthyroid preconception women.

**Methods:**

This retrospective cohort study used data from the National Free Prepregnancy Checkups Project (NFPCP) collected between 2010 and 2020. Female Han Chinese participants aged 20–49 years who had two repeated NFPCP participations with a time interval of 1.5–3.0 years, confirmed non-pregnant status within this duration, and normal thyrotropin levels during their first participation were included for the analysis of thyrotropin abnormalities during the second NFPCP examination. Data were analyzed between June 1 and October 1, 2023.

**Results:**

This study included 186,095 euthyroid women of reproductive age (mean ± SD, 26.72 ± 4.70 years) whose preconception thyrotropin levels were between 0.37 and 4.87 mIU/L. The median follow-up time was 2.13 (IQR, 1.85–2.54) years. A total of 8,497 (4.57%) women developed abnormal thyrotropin, including 4,118 (2.21%) subnormal thyrotropin and 4,379 (2.35%) supranormal thyrotropin. Compared with the reference group (thyrotropin 1.01–2.00 mIU/L), the lower baseline thyrotropin group had greater risk of developing subnormal thyrotropin, and the higher baseline thyrotropin group had greater risk of developing supranormal thyrotropin. Moreover, the restricted cubic spline analysis revealed a U-shaped dose–response association of baseline thyrotropin levels or thyrotropin multiples of the median (MOM) levels against risk of subnormal thyrotropin in the follow-up, and a J-shaped dose–response association against risk of supranormal thyrotropin levels in the follow-up. We further found that baseline thyrotropin outside of 1.43–1.93 mIU/L or baseline thyrotropin MOM outside 0.59–1.36 would hava a higher risk of developing of abnormal thyrotropin.

**Conclusions:**

Both low and high baseline thyrotropin were associated with a significantly increased risk of developing abnormal thyrotropin outcomes. The optimal preconception baseline thyrotropin levels may be between 1.43 mIU/L and 1.93 mIU/L or baseline thyrotropin MoM between 0.59 and 1.36 to minimize progression toward abnormal thyrotropin after 1.5–3.0 years. These findings may help with counseling of preconception thyroid function monitoring.

**Supplementary Information:**

The online version contains supplementary material available at 10.1186/s13044-024-00192-w.

## Background

Thyroid dysfunction is relatively common in women of reproductive age, which may affect maternal reproductive health and fetal development when they conceive successfully [1–4]. Our previous studies reported that both subnormal and supranormal preconception serum thyrotropin levels are associated with adverse pregnancy outcomes including preterm birth, small for gestational age, and perinatal infant death. Abnormal thyrotropin levels are also linked with longer time to pregnancy and higher risk of spontaneous abortion in women aged 20–49 years old [5, 6]. These studies indicate that a thyrotropin level between 0.37 and 2.49 mIU/L may represent the optimal range for preconception women [5, 6].

In consideration of the importance of normal thyroid function for uneventful pregnancy as well as the above-mentioned findings [7–9], preconception thyroid function screening is increasingly recommended by clinicians and gradually accepted by women of reproductive age planning for pregnancy. However, not all women of reproductive age can successfully conceive soon after their evaluation of preconception thyroid function. There are limited studies on the natural change of preconception thyrotropin levels in women of reproductive age. Thus, it is unclear for clinicians and women of reproductive age the frequency of thyrotropin monitoring in euthyroid women identified by the initial examination before they conceive.

Therefore, in this current population-based study, we aimed to investigate the natural progression to abnormal thyrotropin in euthyroid preconception women, as well as identify the optimal thyrotropin range that indicate the lowest risk of developing thyrotropin abnormalities.

## Methods

### Data sources and study population

All participants in this cohort study were from the National Free Prepregnancy Checkups Project (NFPCP), which was a national preconception healthcare service supported by the National Health Commission and the Ministry of Finance of the People’s Republic of China. This project encouraged married couples of reproductive age (20–49 years) to join the project based on their fertility willingness. It would offer free pre-pregnancy eugenics health care services to these couples, including health education, health examinations, risk assessments, and counseling guidance. Based on the assessments of the risk of genetics, reproduction, disease susceptibility, and exposure to environmental toxins, couples were categorized as either the general population or high-risk population, with the latter indicating abnormalities in one or more aspects. For couples planning for pregnancy without identified risk factors, it was recommended to receive regular health education and guidance. However, for couples with identified risk factors, it was advisable to receive further counseling, examination, treatment, and referral. When necessary, it was advised to postpone pregnancy. More detailed information on the design, organization, and implementation of the NFPCP has been described previously [10–12].

Because Han Chinese female participants account for more than 90% of the total population in the NFPCP and to avoid differences in preconception thyrotropin levels among female participants by ethnicity, only female Han Chinese participants who were aged 20–49 years and participated consecutively in the NFPCP from 2010 to 2020 were included in this large-scale retrospective cohort study. The baseline screening was established as the initial NFPCP participation within the period from January 1, 2010, to May 31, 2018, while the follow-up screening represented a subsequent participation occurring within a time interval of 1.5 to 3.0 years. Participants confirmed non-pregnancy during the follow-up period by consistently reporting the same gravidity in the two preconception examinations, alongside recording non-pregnancy during their initial NFPCP involvement.

**Fig. 1:**
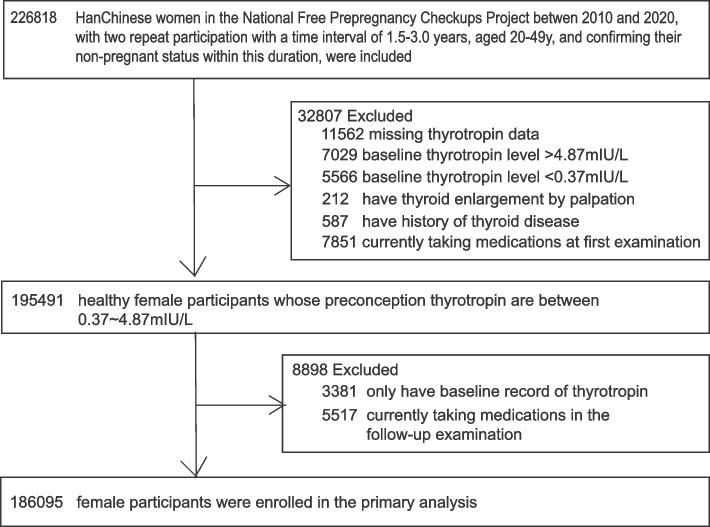
Flowchart of the study cohort selection criteria

Since certain medications could influence thyrotropin levels, participants who self-reported of currently taking medication, including prescription, over-the-counter, or traditional Chinese medicine, to treat any diagnosed disease at the time of the two NFPCP screening were excluded. Moreover, participants with missing or abnormal baseline thyrotropin levels, palpable thyroid enlargement, and history of thyroid disease were excluded. Then, participants without a recorded follow-up thyrotropin were excluded. Finally, 186,095 euthyroid female participants were enrolled in the analysis (Fig. 1).

This study was approved by the Institutional Research Review Board at the National Research Institute for Family Planning in Beijing, China. Written informed consent was provided by every participant before enrollment. This study followed the Strengthening the Reporting of Observational Studies in Epidemiology (STROBE) reporting guidelines.

### Data collection and outcomes

Blood samples and information were collected by trained healthcare staff in local maternal and child service centers or family-planning service institutions. Information on the participants’ demographics, past medication, disease history, obstetric history, dietary nutrition, lifestyle, environmental toxins, psychosocial factors, laboratory tests, and physical examination were recorded in the preconception medical chart.

The outcome was abnormal thyrotropin levels in the follow-up thyrotropin examination, including subnormal thyrotropin and supranormal thyrotropin.

### Serum thyrotropin measurements

Preconception blood samples were collected in the morning from participants after at least 8 h of fasting and immediately sent to local laboratories of maternal and child service centers. Measurement of serum thyrotropin levels were based on the National Guide to Clinical Laboratory Procedures. As all participants came from 2,526 counties of 31 provinces, it was impractical for all laboratories across the different counties to use a uniform thyrotropin detection kit or detection method. Thus, all laboratories within each county consistently used a single type of thyrotropin assay, either electrochemiluminescence immunoassay or enzyme-linked immunosorbent assay kits.

As reported in previous analyses using the same database, a population-specific thyrotropin reference range for preconception thyrotropin levels were established based on the 2.5th and 97.5th percentiles of thyrotropin level of 1,646,039 Han Chinese women who delivered a healthy infant and did not have any of the following conditions: 1) abnormal body mass index (BMI) at baseline; 2) exposure to cigarette smoke or alcohol; 3) history of adverse pregnancy outcome, anemia, diabetes, hypertension, or thyroid disorders; 4) current use of medications. Consistent with our previous study, preconception thyrotropin level of 0.37–4.87 mIU/L was defined as reference range, < 0.37mIU/L was defined as subnormal thyrotropin level, and ≥ 4.88 mIU/L was defined as supranormal thyrotropin level [5].

In addition, to avoid variability or systematic differences among various local laboratories or assays, preconception thyrotropin values were converted to multiples of the median (MOM) for analysis. The thyrotropin MOM value was calculated by dividing the participant’s thyrotropin value by the median thyrotropin value in the reference population of the county where they registered for preconception health examinations.

### Assessment of covariates

Covariates obtained from the baseline preconception examination included age, BMI, parity, education level, alcohol consumption, active smoking, passive smoking status, diabetes, hypertension, and iodine nutrition status. Participants self-reported their birth date, educational attainment, drinking, active smoking and passive smoking status. Age was categorized into five groups (i.e., 20–24.9 years, 25–29.9 years, 30–34.9 years, 35–39.9 years and ≥ 40 years). Technicians measured height and weight to derive BMI. BMI was calculated as weight in kilograms divided by height in square meters (kg/m^2^), while body weight and height were measured with light indoor clothes without shoes and accessories. Underweight, normal weight, overweight and obesity were defined as BMI < 18.5 kg/m^2^, 18.5 kg/m^2^ ≤ BMI ≤ 23.9 kg/m^2^, 24.0 kg/m^2^ ≤ BMI ≤ 27.9 kg/m^2^, and BMI ≥ 28 kg/m^2^, respectively, according to the BMI standard classifications in China. Parity was divided into 2 categories: = 0 or ≥ 1. Higher education level was defined as high school or above. Alcohol consumption was defined as frequently drinking; active smoker was defined as participants currently with at least 1 cigarette a day; whereas passive smoker was defined as involuntary inhalation of smoke from cigarettes or other tobacco products smoked by other people. Diabetes was defined as fasting plasma glucose level of 7.0 mmol/L (126 mg/dL) or higher, or self-reported physician-diagnosed diabetes. Blood pressure was measured on the right arm of the participants using an automated blood pressure monitor after a 5-min rest. Hypertension was defined as systolic blood pressure ≥ 140 mmHg and/or diastolic blood pressure ≥ 90 mmHg or physician-diagnosed hypertension. Iodine nutrition status at the province level were classified into three levels: excessive iodine intake (Jiangsu, Sichuan, Qinghai, Anhui), more than adequate iodine intake (Hebei, Zhejiang, Shanghai, Gansu, Yunnan, Xinjiang, Xizang, Henan, Hunan, Hubei, Inner Mongolia) and adequate iodine intake (Guangdong, Shandong, Guangxi, Fujian, Tianjin, Hainan, Liaoning, Beijing, Guizhou, Shanxi, Ningxia, Jiangxi, Jilin, Shanxi, Heilongjiang).

### Statistical analysis

Mean and standard deviation (SD) were adopted to express continuous variables with normal distribution, and median and interquartile range (IQR) were adopted to express nonnormally distributed variables. Categorical variables were summarized as numbers and percentages. The differences in the characteristics of participants with different thyrotropin levels were tested using Analysis of Variance or the Kruskal–Wallis test for continuous variables, as appropriate, and the chi-square test for categorical variables.

Multivariate-adjusted Cox proportional hazards models were used to estimate hazard ratios (HRs) and 95% confidence intervals (CIs) for the associations between normal thyrotropin levels at baseline and outcomes (including subnormal and supranormal thyrotropin level). Baseline thyrotropin levels were stratified into five groups: 0.37–1.00 mIU/L, 1.01–2.00 mIU/L (referent), 2.01–3.00 mIU/L, 3.01–4.00 mIU/L, 4.01–4.87 mIU/L. In these models, we adjusted for age, BMI, parity, education level, alcohol consumption, passive smoking status, diabetes, hypertension, and iodine nutrition status. Participants (less than 2.5%) with missing covariates data were removed from each model.

To determine an optimal thyrotropin reference range in euthyroid women of reproductive age indicating minimal risk of developing thyrotropin abnormality, the following approach was adopted. Restricted cubic splines (RCS) were constructed to display the fully adjusted relationship of normal thyrotropin level or thyrotropin MOM value with abnormal thyrotropin outcome. To determine the number of knots to use, 3 to 10 knots were assessed, and optimal knots that had the lowest Akaike information criterion were used. The nonlinearity of each dose–response association was tested by Wald statistics. The covariates in dose–response association analysis models were the same as those in the previous Cox proportional hazards regression model. The initial thyrotropin values that corresponded to the lowest statistically significant HR > 1.00 was determined as possible cutoffs for the optimal thyrotropin range. Statistical analysis was performed using R software version 4.0.3. All statistical tests were two-sided, and *P* < 0.05 was considered statistically significant. Data were analyzed between June 1 and October 1, 2023.

## Results

### Cohort characteristics

A total of 186,095 euthyroid women of reproductive age whose preconception thyrotropin were between 0.37 and 4.87 mIU/L at first examination were enrolled in the analysis. The median time interval between the two NFPCP examinations was 2.13 years (IQR, 1.85–2.54) (Fig. 1). Demographics of these euthyroid female participants are presented in the Table 1. The mean age was 26.7 ± 4.7 years old and more than 80% were less than 30 years old. The mean BMI was 21.6 ± 3.0 kg/m^2^ with 70.6% between 18.5 and 23.9 kg/m^2^. Most participants had a parity ≥ 1, received high school education or above, no alcohol consumption, no active smoking, no passive smoke exposure, and no diabetes or hypertension history. Of note, all participants were from iodine sufficient regions.

**Table 1:** Characteristics of participants according to baseline preconception thyrotropin levels^a^

Characteristic	Overall (*n *= 186095)	Thyrotropin, median (IQR) mIU/L	Maternal preconception thyrotropin levels, mIU/L					
			0.37-1.00 (*n *= 32678)	1.01-2.00 (*n *= 78141)	2.01-3.00 (*n *= 49719)	3.01-4.00 (*n *- 19391)	4.01-4.87 (*n *= 6166)	*P *value
Thyrotropin, median (IQR), mIU/L		1.75 [1.19, 2.48]	0.77 [0.60, 0.90]	1.46 [1.24, 1.72]	2.40 [2.18, 2.64]	3.35 [3.16, 3.62]	4.30 [4.13, 4.53]	< 0.001^b^
Stratified for age (years)								< 0.001^c^
20-24.9	78621(42.2)	1.72 [1.17, 2.45]	14340 (43.9)	33061 (42.3)	20682 (41.6)	8051 (41.5)	2487 (40.3)	
25-29.9	70921(38.1)	1.77 [1.20, 2.50]	12056 (36.9)	29575 (37.8)	19493 (39.2)	7433 (38.3)	2364 (38.3)	
30-34.9	23678 (12.7)	1.73 [1.19, 2.48]	4169 (12.8)	10014 (12.8)	6198 (12.5)	2488 (12.8)	809 (13.1)	
35-39.9	9216( 5.0)	1.75 [1.20, 2.48]	1545 ( 4.7)	3977 ( 5.1)	2380 ( 4.8)	980 ( 5.1)	334 ( 5.4)	
> 40	3659 ( 2.0)	1.82 [1.24, 2.61]	568 ( 1.7)	1514 ( 1.9)	966 ( 1.9)	439 ( 2.3)	172 ( 2.8)	
Stratified for BMI (kg/m2)								< 0.001^c^
< 18.5	22243 (12.0)	1.73 [1.16, 2.46]	4141 (12.7)	9299 (11.9)	5817 (11.7)	2257 (11.6)	729 (11.8)	
18.5-23.9	131433 (70.6)	1.74 [1.20, 2.47]	22868 (70.0)	55559 (71.1)	35366 (71.1)	13415 (69.2)	4225 (68.5)	
24.0-27.9	25184 (13.5)	1.76 [1.17, 2.51]	4529 (13.9)	10325 (13.2)	6580 (13.2)	2829 (14.6)	921 (14.9)	
> 28.0	6534 ( 3.5)	1.83 [1.24, 2.59]	1019 ( 3.1)	2658 ( 3.4)	1751 ( 3.5)	834 ( 4.3)	272 ( 4.4)	
Missing data	701 ( 0.4)	1.77 [1.20, 2.37]	121 ( 0.4)	300 ( 0.4)	205 ( 0.4)	56 ( 0.3)	19 ( 0.3)	
Stratified for Parity								< 0.001^c^
0	65662 (35.3)	1.75 [1.20, 2.45]	11369 (34.8)	27991 (35.8)	17826 (35.9)	6542 (33.7)	1934 (31.4)	
≥ 1	108440 (58.3)	1.76 [1.20, 2.50]	18794 (57.5)	45159 (57.8)	29010 (58.3)	11654 (60.1)	3823 (62.0)	
Missing data	11993 ( 6.4)	1.65 [1.10, 2.40]	2515 ( 7.7)	4991 ( 6.4)	2883 ( 5.8)	1195 ( 6.2)	409 ( 6.6)	
Stratified for education								< 0.001^c^
High school or above	126616 (68.0)	1.75 [1.19, 2.49]	22356 (68.4)	52553 (67.3)	33863 (68.1)	13525 (69.7)	4319 (70.0)	
Primary school or below	54989 (29.5)	1.74 [1.20, 2.46]	9515 (29.1)	23498 (30.1)	14794 (29.8)	5475 (28.2)	1707 (27.7)	
Missing data	4490 ( 2.4)	1.65 [1.18, 2.36]	807 ( 2.5)	2090 ( 2.7)	1062 ( 2.1)	391 ( 2.0)	140 ( 2.3)	
Stratified for alcohol consumption								< 0.001^c^
No	180000 (99.3)	1.74 [1.19, 2.47]	31704 (97.0)	75681 (96.9)	47997 (96.5)	18683 (96.3)	5935 (96.3)	
Yes	5201 ( 0.3)	1.83 [1.23, 2.58]	829 ( 2.5)	2102 ( 2.7)	1462 ( 2.9)	598 ( 3.1)	210 ( 3.4)	
Missing data	894 ( 0.4)	1.83 [1.21, 2.50]	145 ( 0.4)	358 ( 0.5)	260 ( 0.5)	110 ( 0.6)	21 ( 0.3)	
Stratified for active smoking								< 0.757^c^
No	184778 (99.4)	1.90 [1.19, 2.48]	32473 (99.4)	77622 (99.3)	49394 (99.3)	19264(99.3)	6134 (99.5)	
Yes	536 (0.3)	1.93 [1.23, 2.51]	81 ( 0.2)	233 ( 0.3)	152 ( 0.3)	55 ( 0.3)	15 ( 0.2)	
Missing data	672 ( 0.4)	1.87 [1.19, 2.36]	124 ( 0.4)	286 ( 0.4)	173 ( 0.3)	72 ( 0.4)	17 ( 0.3)	
Stratified for secondhand smoke								< 0.001^c^
No	165255 (88.8)	1.74 [1.19, 2.46]	29183 (89.3)	69701 (89.2)	43981 (88.5)	17006 (87.7)	5384 (87.3)	
Yes	20101 (10.8)	1.81 [1.22, 2.56]	3363 (10.3)	8126 (10.4)	5546 (11.2)	2303 (11.9)	763 (12.4)	
Missing data	739 ( 0.4)	1.75 [1.20, 2.45]	132 ( 0.4)	314 ( 0.4)	192 ( 0.4)	82 ( 0.4)	19 ( 0.3)	
Stratified for diabetes								< 0.001^c^
No	182400 (98.0)	1.75 [1.20, 2.48]	31995 (97.9)	76607 (98.0)	48769 (98.1)	18992 (97.9)	6037 (97.9)	
Yes	2166 ( 1.2)	1.67 [1.11, 2.41]	456 ( 1.4)	882 ( 1.1)	552 ( 1.1)	215 ( 1.1)	61 ( 1.0)	
Missing data	1529 ( 0.8)	1.80 [1.24, 2.62]	227 ( 0.7)	652 ( 0.8)	398 ( 0.8)	184 ( 0.9)	68 ( 1.1)	
Stratified for hypertension								< 0.001^c^
No	117793 (63.3)	1.72 [1.18, 2.45]	21129 (64.7)	49883 (63.8)	30877 (62.1)	12129 (62.5)	3775 (61.2)	
Yes	1817 ( 1.0)	1.86 [1.22, 2.63]	299 ( 0.9)	720 ( 0.9)	478 ( 1.0)	231 ( 1.2)	89 ( 1.4)	
Missing data	66485 (35.7)	1.78 [1.20, 2.50]	11250 (34.4)	27538 (35.2)	18364 (36.9)	7031 (36.3)	2302 (37.3)	
Stratified for environmental iodine status								< 0.001^c^
Adequate iodine	71800 (38.6)	1.73 [1.18, 2.46]	12708 (38.9)	30595 (39.2)	18990 (38.2)	7239 (37.3)	2268 (36.8)	
More than adequate iodine	94630 (50.9)	1.73 [1.18, 2.45]	17100 (52.3)	39594 (50.7)	25402 (51.1)	9572 (49.4)	2962 (48.0)	
Excess iodine	19665 ( 10.6)	1.88 [1.27, 2.65]	2870 ( 8.8)	7952 (10.2)	5327 (10.7)	2580 (13.3)	936 (15.2)	

*Abbreviations*: *BMI* body mass index (calculated as weight in kilograms divided by height in meters squared), *IQR* interquartile range

^a^Data presented as number (percentage) unless otherwise indicated

^b^Differences in the characteristics of participants with different thyrotropin levels were assessed using Kruskal–Wallis test for continuous variables

^c^Differences in categorical variables were evaluated using chi-square test

### Preconception serum thyrotropin levels at baseline

The median preconception thyrotropin level among 186,095 euthyroid female participants at baseline was 1.75 (IQR, 1.19–2.48) mIU/L. These participants were stratified into five levels according to their baseline thyrotropin. There were 32,678 (17.56%), 49,719 (26.72%), 19,391 (10.42%), and 6,166 (3.31%) participants with baseline thyrotropin of 0.37–1.00 mIU/L, 2.01–3.00 mIU/L, 3.01–4.00 mIU/L, and 4.01–4.87 mIU/L, respectively. Of note, women with preconception thyrotropin levels between 1.01 and 2.00 mIU/L accounted for 41.99% of participants (*n* = 78,141) (Table 1).

Among participants aged between 20 and 24.9 years, there were gradually fewer participants as the preconception thyrotropin level increased, but this trend was inversed among participants aged over 40 years. Moreover, the proportion of participants who had BMI ≥ 28.0 kg/m^2^, parity ≥ 1, hypertension, alcohol consumption, exposure to second-hand smoke, or from regions with iodine excessive status, gradually increased as the preconception thyrotropin level increased (Table 1).

### Natural change of preconception serum thyrotropin in the follow-up examination

**Fig. 2:**
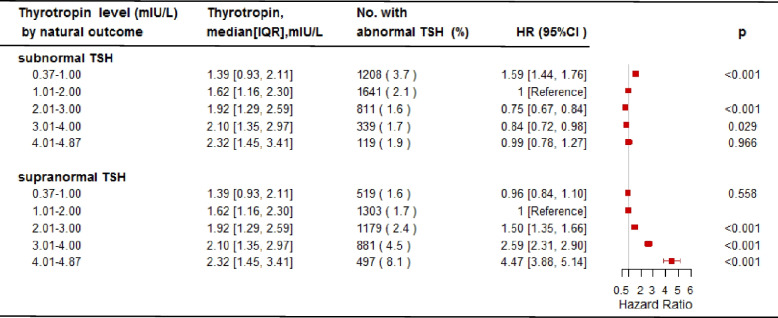
Hazard ratios (HR) of abnormal thyrotropin levels according to baseline thyrotropin levels. Subnormal thyrotropin was defined as thyrotropin < 0.37 mIU/L and supranormal thyrotropin was defined as thyrotropin ≥ 4.88 mIU/L. First column, thyrotropin level at baseline (mIU/L); second column, median of follow-up thyrotropin level (mIU/L); third column, number of participants who developed abnormal thyrotropin in the follow-up; fourth column, HRs of developing abnormal thyrotropin compared to the reference group (thyrotropin 1.01–2.00 mIU/L). Cox proportional hazard regression model was adjusted for age, body mass index, parity, education level, alcohol consumption, passive smoking, diabetes, hypertension, and environmental iodine nutrition status. Abbreviations: TSH, thyrotropin; IQR, interquartile range; HR, hazard ratio; CI, confidence interval

In the baseline thyrotropin subgroups of 0.37–1.00 mIU/L and 1.01–2.00 mIU/L, the median follow-up thyrotropin level increased from baseline, from initial thyrotropin 0.77 (IQR, 0.60, 0.90) mIU/L to 1.39 (0.93, 2.11) mIU/L and from 1.46 (1.24, 1.72) mIU/L to 1.62 (1.16, 2.30) mIU/L, respectively. In contrast, the median follow-up thyrotropin decreased from 2.40 (IQR, 2.18, 2.64) mIU/L to 1.92 (1.29, 2.59) mIU/L, from 3.35 (3.16, 3.62) mIU/L to 2.10 (1.35, 2.97) mIU/L, and from 4.30 (4.13, 4.53) mIU/L to 2.32 (1.45, 3.41) mIU/L in baseline thyrotropin subgroups of 2.01–3.00 mIU/L, 3.01–4.00 mIU/L, and 4.01–4.87 mIU/L, respectively (Table 1, Fig. 2).

### Risk of developing thyrotropin abnormality according to the baseline thyrotropin levels

A total of 4,118 (2.21%) participants developed subnormal thyrotropin (thyrotropin < 0.37 mIU/L) and 4,379 (2.35%) participants developed supranormal thyrotropin (thyrotropin ≥ 4.88 mIU/L) among 186,095 euthyroid female participants. Compared to the reference group (thyrotropin 1.01–2.00 mIU/L), baseline thyrotropin subgroup 0.37–1.00 mIU/L had greater risk of developing subnormal thyrotropin (HR, 1.59; 95% CI, 1.44, 1.76), whereas baseline thyrotropin subgroups 2.01–3.00 mIU/L and 3.01–4.00 mIU/L had lower risk of developing subnormal thyrotropin (HR, 0.75 [95% CI, 0.67, 0.84] and HR, 0.84 [95% CI, 0.72, 0.98], respectively). However, baseline thyrotropin subgroup 4.01–4.87 mIU/L had no change in the risk towards later occurrence of subnormal thyrotropin (HR, 0.99 [95% CI, 0.78, 1.27]). In contrast, baseline thyrotropin subgroups between 2.01 and 4.87 mIU/L had greater risk of developing supranormal thyrotropin (2.01–3.00 mIU/L: HR, 1.50 [95% CI, 1.35, 1.66]; 3.01–4.00 mIU/L: HR, 2.59 [95% CI, 2.31, 2.90]; 4.01–4.87 mIU/L: HR, 4.47 [95% CI, 3.88, 5.14]), while there was no longer a significant effect on developing supranormal thyrotropin when baseline thyrotropin levels were limited to 0.37–1.00 mIU/L (HR, 0.96 [95% CI, 0.84, 1.10]) (Fig. 2). Furthermore, similar results were observed in sensitivity analysis after excluding participants with potential risk factors that could impact fertility willingness at the initial NFPCP examination (Supplemental Fig. 1, Supplemental Fig. 2).

### Dose–response association between baseline thyrotropin levels or thyrotropin MoM levels and development of thyrotropin abnormality in the follow-up

**Fig. 3:**
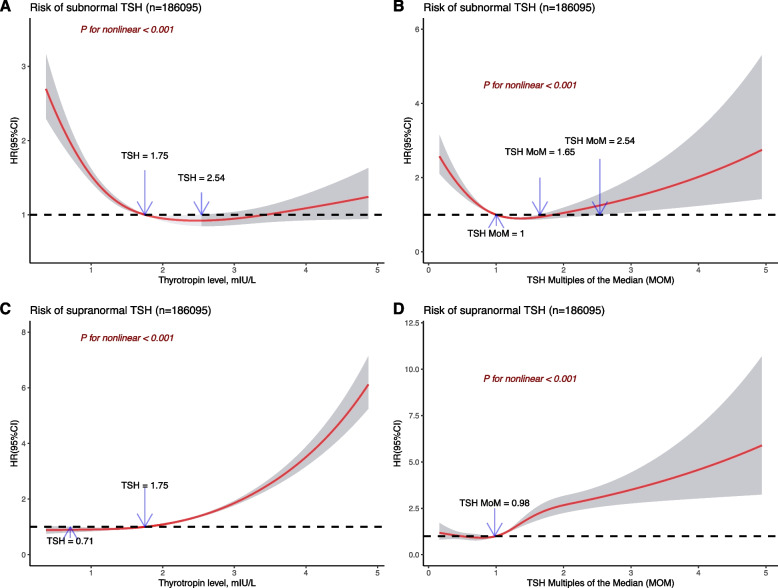
Dose–response association between baseline thyrotropin level or thyrotropin MOM and risk of subnormal or supranormal thyrotropin. Cox proportional hazard regression model was adjusted for age, body mass index, parity, education level, alcohol consumption, passive smoking, diabetes, hypertension, and iodine nutrition status. Reference values were thyrotropin 1.75 mIU/L (**A**, **C**) and thyrotropin MOM 1.00 (**B**, **D**). Black curves indicate HR estimate; shaded areas indicate 95% confidence intervals. Abbreviations: TSH, thyrotropin; MOM, multiples of the median; HR, hazard ratios. Note: The specific number of knots of each model: **A**: 3; **B**:5; **C**:4; **D**:3

RCS analysis revealed a U-shaped dose–response association of baseline thyrotropin levels or thyrotropin MoM with risk of follow-up subnormal thyrotropin (χ^2^ = 100.56; nonlinear *P* < 0.001 or χ^2^ = 58.95; nonlinear *P* < 0.001, respectively), suggesting that baseline thyrotropin outside of 1.75–2.54 mIU/L would have a higher risk of developing subnormal thyrotropin in the follow-up thyrotropin examination (Fig. 3A, B).

The same analysis revealed a J-shaped dose–response association of baseline thyrotropin levels or thyrotropin MoM with risk of follow-up supranormal thyrotropin (χ^2^ = 46.51; nonlinear *P* < 0.001 or χ^2^ = 31.92; nonlinear *P* < 0.001, respectively), indicating that the risk of developing supranormal thyrotropin in the follow-up thyrotropin examination would be the lowest when baseline thyrotropin is around 1.75mIU/L, and then increase as the baseline thyrotropin rises (Fig. 3C, D).

**Fig. 4:**
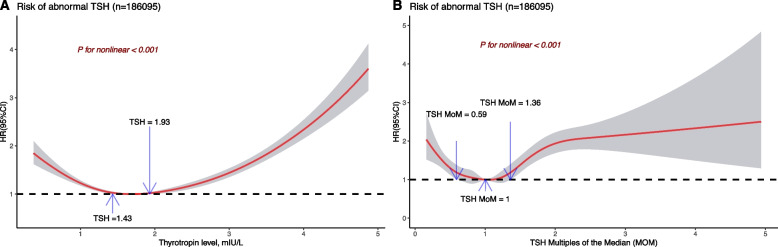
Dose–response association between baseline thyrotropin level or thyrotropin MOM and risk of developing abnormal thyrotropin. Cox proportional hazard regression model was adjusted for age, body mass index, parity, education level, alcohol consumption, passive smoking, diabetes, hypertension, and environmental iodine nutrition status. Reference values were thyrotropin 1.75 mIU/L (**A**) and thyrotropin MOM 1.00 (**B**). Black curves indicate HR estimate; shaded areas indicate 95% confidence intervals. Abbreviations: TSH, thyrotropin; MOM, multiples of the median; HR, hazard ratios. Note: The specific number of knots of each model: **A**: 4; **B**:7

To find the optimal thyrotropin range with minimal risk for developing thyrotropin abnormality several years later in the follow-up, we analyzed the baseline thyrotropin levels in euthyroid women of reproductive age and the risk of thyrotropin abnormality in the follow-up examination. U-shaped dose–response associations were found between baseline thyrotropin levels and risk of thyrotropin abnormality (χ2 = 100.56; nonlinear *P* < 0.001) and between baseline thyrotropin MoM and risk of thyrotropin abnormality (χ2 = 94.54; nonlinear *P* < 0.001). Baseline thyrotropin between 1.43 and 1.93 mIU/L represented an optimal preconception range for women of reproductive age, as it showed the minimal progression toward abnormal thyrotropin in the follow-up thyrotropin examination. Baseline thyrotropin MoM between 0.59 and 1.36 could be considered as the optimal range (Fig. 4A, B).

Furthermore, we excluded initial NFPCP participants who would be considered as having high-risk pregnancy if they conceived successfully and found similar trends in the analysis of dose–response association between baseline thyrotropin levels or thyrotropin MoM values and the risk of thyrotropin abnormality in the follow-up (Supplemental Fig. 3). In this subgroup, the optimal thyrotropin range with minimal risk for developing thyrotropin abnormality in the follow-up closely matched that of the overall cohort (Supplemental Fig. 4).

## Discussion

In this large, nationwide, population-based cohort study of more than 180,000 euthyroid women of reproductive age, we observed that a total of 8,497 (4.57%) women developed abnormal thyrotropin in the follow-up examination, including 4,118 (2.21%) women who developed subnormal thyrotropin and 4,379 (2.35%) women who developed supranormal thyrotropin. Compared with the reference group (thyrotropin 1.01–2.00 mIU/L), lower baseline thyrotropin had greater risk of developing subnormal thyrotropin, and higher baseline thyrotropin had greater risk of developing supranormal thyrotropin. The baseline thyrotropin levels or thyrotropin MoM showed a U-shaped dose–response association with the risk of subnormal thyrotropin in the follow-up, and a J-shaped dose–response association with the risk of supranormal thyrotropin in the follow-up. Baseline thyrotropin levels outside of 1.43–1.93 mIU/L or thyrotropin MoM outside of 0.59–1.36 would hava a higher risk of developing abnormal thyrotropin in the follow-up of 1.5–3.0 years.

Several studies have investigated the natural course of thyroid function in non-euthyroid populations. Lazer et al. found that initial normal or slightly elevated thyrotropin levels in a pediatric cohort were likely to remain normal or spontaneously normalize without any intervention over five years. Children with initial thyrotropin level > 7.5 mIU/L, particularly girls, were at a greater risk for sustained thyrotropin abnormality [13]. Other studies investigated adult patients with slightly abnormal thyroid function have showed that around 0.5% ~ 5.3% patients with subclinical hyperthyroidism would develop into hyperthyroidism and 13.3% ~ 36% would spontaneously go into remission [14–17]; and approximately 2 ~ 6% patients with subclinical hypothyroidism would develop into overt hypothyroidism and about 60% of patients with slightly elevated thyrotropin would achieve spontaneous remission [18–20]. However, little knowledge has been obtained regarding the natural change of thyroid function in euthyroid populations, especially in women of reproductive age who have pregnancy intention. Therefore, we performed the present study to add novel information into this area.

According to the analysis on baseline thyrotropin levels, age, BMI, parity, education level, alcohol consumption, passive smoking, diabetes, hypertension, and iodine nutrition status of the regions were associated with thyrotropin levels. Thus, these factors were adjusted in the Cox proportional hazards models when we further analyzed the association between initial thyrotropin and follow-up thyrotropin levels. Previous studies in pregnant populations have revealed an association between active smoking and thyrotropin levels [21, 22]. However, our study did not yield statistically significant evidence (*P* = 0.757) indicating an impact of active smoking on thyrotropin levels. This observation is probably attributed to the relatively small proportion of active smokers in our sample, accounting for only 0.3% of the total sample. One important and meaningful finding in our study is that among euthyroid women of reproductive age, new onset of thyrotropin abnormality after 1.5–3.0 years is uncommon and associated with baseline thyrotropin levels. Based on the RCS analysis, baseline thyrotropin of 1.43–1.93 mIU/L was the optimal range with the lowest risk of abnormal thyrotropin after 1.5–3.0 years. This range is narrower than the optimal range for adults that we reported previously [23]. In that study, 2,727 initially euthyroid Chinese individuals were followed up for 5 years to observe the incidence of abnormal thyrotropin. Results showed that baseline thyrotropin below 1.0 mIU/L presented higher risk of developing subnormal thyrotropin, while baseline thyrotropin above 1.9 mIU/L had greater likelihood to develop supranormal thyrotropin; an initial thyrotropin between 1.0 and 1.9 mIU/L represented an optimal interval to minimize progression toward abnormal thyrotropin in 5 years [23]. This slightly different findings between the two studies may be attributed to the following reasons: 1) Subjects are different- the previous study enrolled inhabitants with age > 13 years, including adolescent and adult men and women, while the current study solely focused on women of reproductive age between 20 and 49 years. 2) Length of follow-up is different-5 years in the previous study and 1.5–3.0 years in the current study. 3) Methods for identifying the optimal range are different– the previous study roughly defines the range by two separate graphs showing thyrotropin-cumulative incidence association for subnormal and supranormal thyrotropin, whereas this study defined the optimal range by the RCS analysis on the association of baseline thyrotropin and the risk of thyrotropin abnormality in follow-up.

The identification of optimal range for thyrotropin may have clinical implications. It may be adequate to reduce the frequency of preconception thyrotropin function monitoring in women of reproductive age with thyrotropin level 1.43–1.93 mIU/L or thyrotropin MoM value 0.59–1.36 at the initial preconception screening. This can help with decision making in preconception management and save medical costs. Yet, the best strategy and interval for preconception thyrotropin monitoring in women of reproductive age remain unclear. More importantly, no study has elucidated whether certain interventions could reduce the risk of developing abnormal thyrotropin in women with reproductive age whose preconception thyrotropin levels are close to the lower limit or upper limit of the reference range, and how to intervene.

The strengths of the present study include firstly, this is a follow-up study in a large population-based cohort of more than 180,000 euthyroid women of reproductive age; and secondly, both thyrotropin levels and thyrotropin MoM were used to define the optimal range by RCS analysis, the latter could avoid variability or systematic differences among various local laboratories or assays. However, there were several limitations in this study. First, the thyrotropin level for each participant at baseline and follow-up were limited to single-test results, making it challenging to eliminate the influence of fluctuating test values on the study results. The absence of repeated measurements may increase the susceptibility to bias in the study results, particularly for the recommendation of clinical reference range, representing a common limitation in observational studies. Second, thyroid autoantibodies were not analyzed in this study due to lack of data, it is an obvious limitation in this study because previous studies had reported that thyroid autoantibodies were significantly associated with development of thyroid dysfunction and pregnancy outcomes [24, 25]. Third, iodine nutrition is a key determinant of thyroid dysfunction risk [26–28], but we could not compare differences in population with iodine deficiency and iodine excess because most regions in China are iodine-sufficient since the implementation of universal salt iodization policy in China [29]. Thus, the results of this study may not be generalizable to other populations whose iodine nutrition, especially if iodine deficient, is different from China. Fourth, this study was derived from the NFPCP project, which inevitably encounter the challenge of selection bias, such as majority of the participants were from rural regions and the interval time of participation may be influenced not only by individual fertility willingness but also by risk factors that render pregnancy unsuitable. To compensate for this limitation, we conducted post-hoc analysis and excluded participants with risk factors for pregnancy and found that the results remained consistent with the main findings. Nonetheless, these limitations have a negligible impact on the final conclusion of this study, but they are worth further exploring in the future.

## Conclusion

In this retrospective cohort study, we found that 4.57% of euthyroid women of reproductive age developed thyroid dysfunction in the follow-up thyrotropin examination, including 2.21% of women developed subnormal thyrotropin and 2.35% of women developed supranormal thyrotropin. Both low and high preconception baseline thyrotropin levels were associated with significantly increased risk of developing abnormal thyrotropin outcomes. The optimal baseline thyrotropin levels may be between 1.43 and 1.93 mIU/L or baseline thyrotropin MoM between 0.59 and 1.36 to minimize progression toward abnormal thyrotropin after 1.5–3.0 years. These findings may help with counseling in preconception thyroid function monitoring.

### Supplementary Information

**Additional file 1: Supplemental Figure 1.** Flowchart of the study cohort selection criteria with excluding individuals who were not suitable for pregnancy at baseline.

**Additional file 2: Supplemental Figure 2. ** Analysis of hazard ratios of abnormal thyrotropin levels according to baseline thyrotropin levels after excluding individuals who were not suitable for pregnancy at baseline. Fist column, thyrotropin level in baseline; second column, median level of follow-up thyrotropin; third column, number of participants who developed abnormal TSH in the follow-up; fourth column, hazard ratios of abnormal thyrotropin levels in other groups comparing with reference group (thyrotropin level between 1.01 and 2.00 mIU/L). Cox proportional hazard regression model was adjusted for age, body mass index, parity, education, alcohol consumption, passive smoking, and environmental iodine status. Abbreviations: TSH, thyrotropin; IQR, interquartile range; HR, hazard ratios; CI, Confidence interval.

**Additional file 3: Supplemental Figure 3.** Analysis of dose-response association between baseline thyrotropin or thyrotropin MOM and risk of subnormal or supranormal thyrotropin after excluding individuals who were not suitable for pregnancy at baseline. Cox proportional hazard regression model was adjusted for age, body mass index, parity, education, alcohol consumption, passive smoking, and environmental iodine status. Reference values were 1.75 mIU/L thyrotropin (A, C) and 1.00 MOM thyrotropin (B, D). Black curves indicate risk estimate; shaded areas, 95% CIs. Abbreviations: TSH, thyrotropin; MOM, multiples of the median; HR, hazard ratios. The specific number of knots of each model: A: 5; B:4; C:3; D:4.

**Additional file 4: Supplemental Figure 4.** Analysis of dose-response association between baseline thyrotropin or thyrotropin MOM and risk of developing abnormal thyrotropin after excluding individuals who were not suitable for pregnancy at baseline. Cox proportional hazard regression model was adjusted for age, body mass index, parity, education, alcohol consumption, passive smoking, and environmental iodine status. Reference values were 1.75 mIU/L thyrotropin (A) and 1.00 MOM thyrotropin (B). Black curves indicate risk estimate; shaded areas, 95% CIs. Abbreviations: TSH, thyrotropin; MOM, multiples of the median; HR, hazard ratios. The specific number of knots of each model: A: 5; B:7.

## Data Availability

The datasets and materials analyzed in this study are available from the corresponding author on reasonable request.

## Abbreviations

BMI: Body mass index
CI: Confidence interval
HR: Hazard ratio
IQRs: Interquartile ranges
MOM: Multiples of the median
NFPCP: National Free Prepregnancy Checkups Project
RCS: Restricted cubic spline
SD: Mean values
STROBE: Strengthening the Reporting of Observational Studies in Epidemiology
