# Supplementary material for: Evaluating the progression to abnormal thyrotropin in euthyroid preconception women: a population-based study
Source: Thyroid Res. 2024 Mar 11;17:5. doi: 10.1186/s13044-024-00192-w (PMC10926655; doi:10.1186/s13044-024-00192-w)
Supplement: Supplementary file 1 — Additional file 1: Supplemental Figure 1. Flowchart of the study cohort selection criteria with excluding individuals who were not suitable for pregnancy at baseline. [file 13044_2024_192_MOESM1_ESM.pdf]

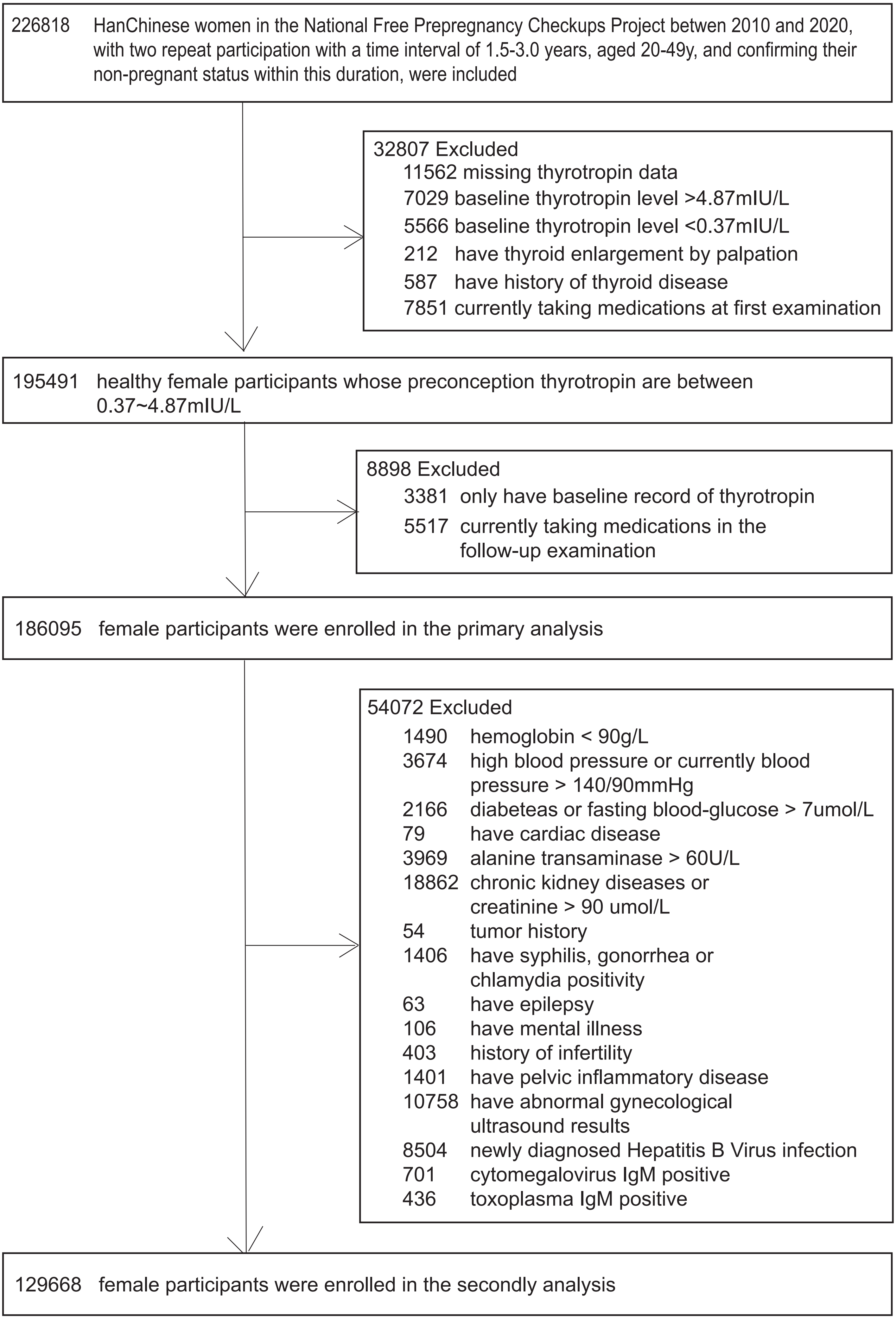

226818 HanChinese women in the National Free Prepregnancy Checkups Project betwen 2010 and 2020, with two repeat participation with a time interval of 1.5-3.0 years, aged 20-49y, and confirming their non-pregnant status within this duration, were included
32807 Excluded
11562 missing thyrotropin data
7029 baseline thyrotropin level >4.87mIU/L
5566 baseline thyrotropin level <0.37mIU/L
212 have thyroid enlargement by palpation
587 have history of thyroid disease
7851 currently taking medications at first examination
195491 healthy female participants whose preconception thyrotropin are between 0.37~4.87mIU/L
8898 Excluded
3381 only have baseline record of thyrotropin
5517 currently taking medications in the follow-up examination
186095 female participants were enrolled in the primary analysis
54072 Excluded
1490 hemoglobin < 90g/L
3674 high blood pressure or currently blood pressure > 140/90mmHg
2166 diabeteas or fasting blood-glucose > 7umol/L
79 have cardiac disease
3969 alanine transaminase > 60U/L
18862 chronic kidney diseases or creatinine > 90 umol/L
54 tumor history
1406 have syphilis, gonorrhea or chlamydia positivity
63 have epilepsy
106 have mental illness
403 history of infertility
1401 have pelvic inflammatory disease
10758 have abnormal gynecological ultrasound results
8504 newly diagnosed Hepatitis B Virus infection
701 cytomegalovirus IgM positive
436 toxoplasma IgM positive
129668 female participants were enrolled in the secondly analysis
