# Supplementary figures and images for: Evaluating the progression to abnormal thyrotropin in euthyroid preconception women: a population-based study
Source: Thyroid Res. 2024 Mar 11;17:5. doi: 10.1186/s13044-024-00192-w (PMC10926655; doi:10.1186/s13044-024-00192-w)

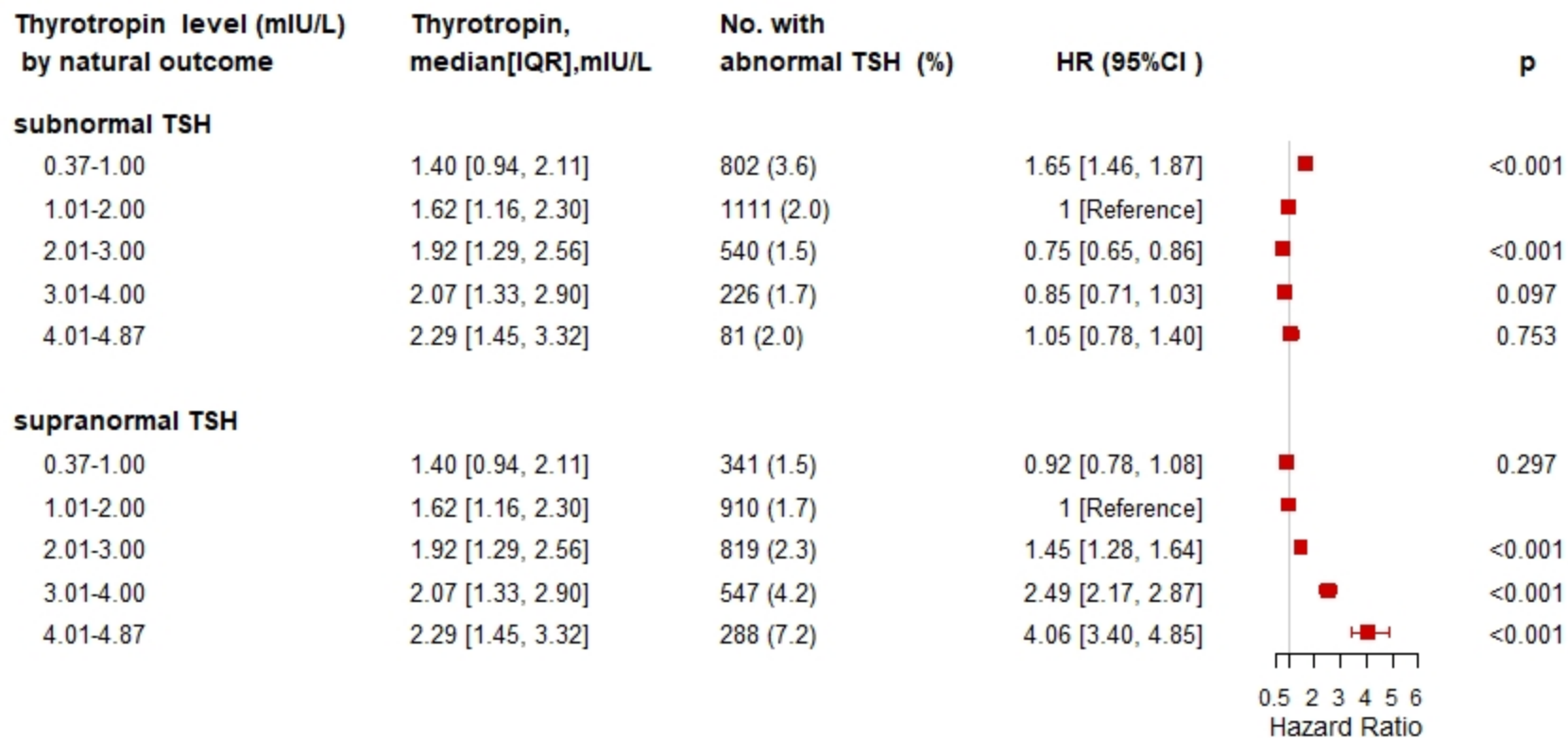

Supplement: Supplementary file 2 — Additional file 2: Supplemental Figure 2. Analysis of hazard ratios of abnormal thyrotropin levels according to baseline thyrotropin levels after excluding individuals who were not suitable for pregnancy at baseline. Fist column, thyrotropin level in baseline; second column, median level of follow-up thyrotropin; third column, number of participants who developed abnormal TSH in the follow-up; fourth column, hazard ratios of abnormal thyrotropin levels in other groups comparing with reference group (thyrotropin level between 1.01 and 2.00 mIU/L). Cox proportional hazard regression model was adjusted for age, body mass index, parity, education, alcohol consumption, passive smoking, and environmental iodine status. Abbreviations: TSH, thyrotropin; IQR, interquartile range; HR, hazard ratios; CI, Confidence interval. [file 13044_2024_192_MOESM2_ESM.pdf]

**A** Risk of abnormal TSH (n=129668)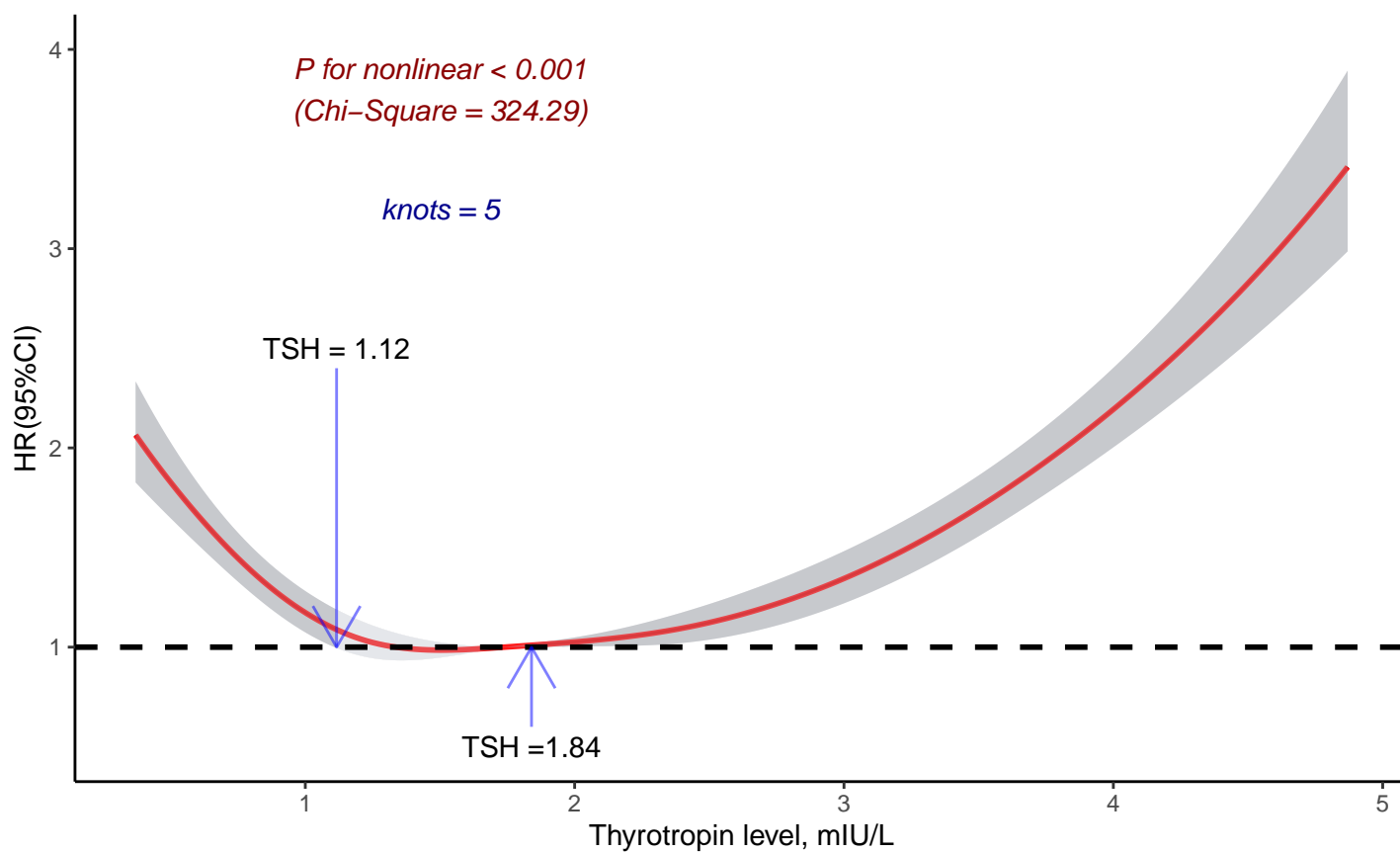**B** Risk of abnormal TSH (n=129668)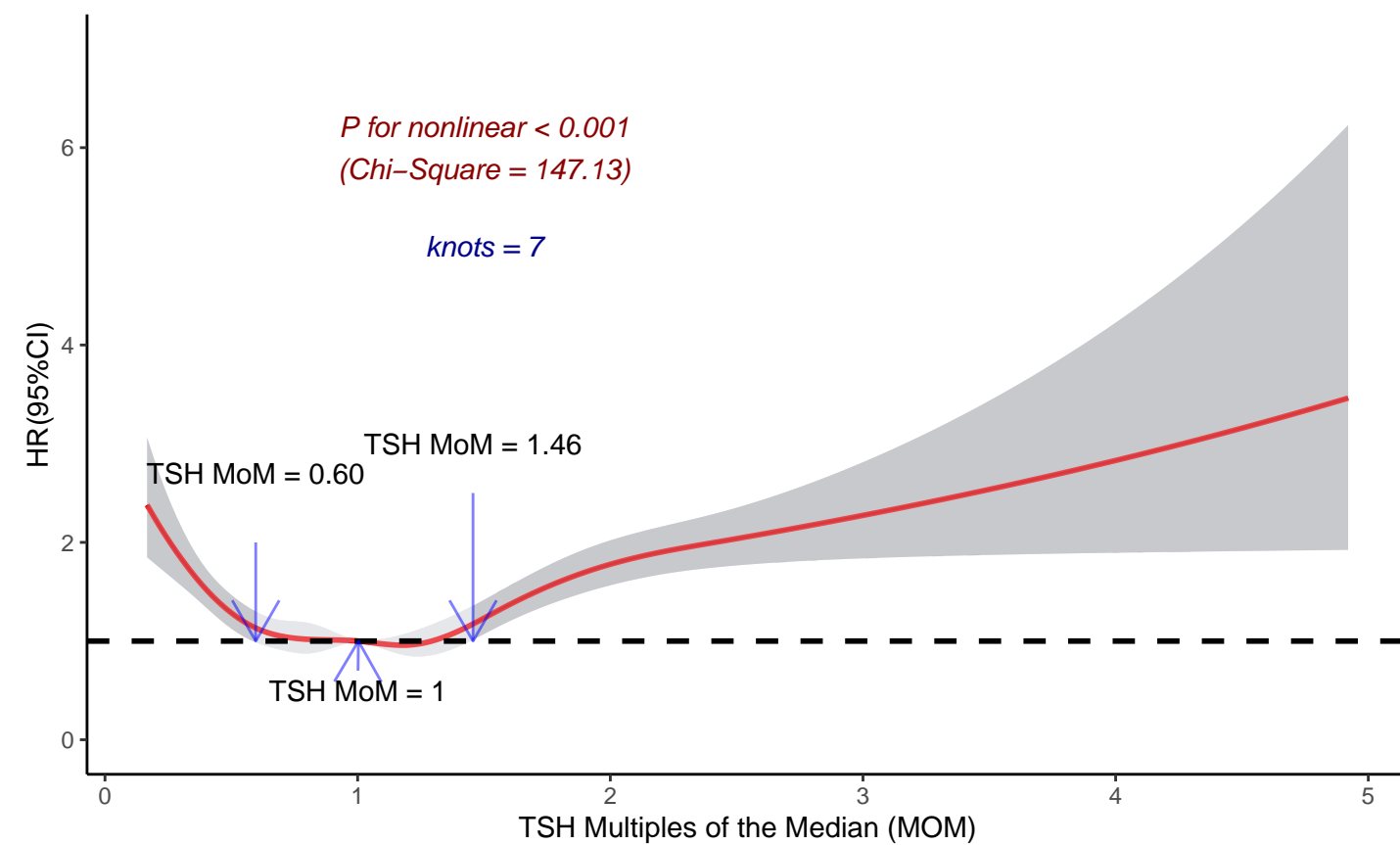

Supplement: Supplementary file 4 — Additional file 4: Supplemental Figure 4. Analysis of dose-response association between baseline thyrotropin or thyrotropin MOM and risk of developing abnormal thyrotropin after excluding individuals who were not suitable for pregnancy at baseline. Cox proportional hazard regression model was adjusted for age, body mass index, parity, education, alcohol consumption, passive smoking, and environmental iodine status. Reference values were 1.75 mIU/L thyrotropin (A) and 1.00 MOM thyrotropin (B). Black curves indicate risk estimate; shaded areas, 95% CIs. Abbreviations: TSH, thyrotropin; MOM, multiples of the median; HR, hazard ratios. The specific number of knots of each model: A: 5; B:7. [file 13044_2024_192_MOESM4_ESM.pdf]
