# Supplementary material for: Evaluating the progression to abnormal thyrotropin in euthyroid preconception women: a population-based study
Source: Thyroid Res. 2024 Mar 11;17:5. doi: 10.1186/s13044-024-00192-w (PMC10926655; doi:10.1186/s13044-024-00192-w)
Supplement: Supplementary file 3 — Additional file 3: Supplemental Figure 3. Analysis of dose-response association between baseline thyrotropin or thyrotropin MOM and risk of subnormal or supranormal thyrotropin after excluding individuals who were not suitable for pregnancy at baseline. Cox proportional hazard regression model was adjusted for age, body mass index, parity, education, alcohol consumption, passive smoking, and environmental iodine status. Reference values were 1.75 mIU/L thyrotropin (A, C) and 1.00 MOM thyrotropin (B, D). Black curves indicate risk estimate; shaded areas, 95% CIs. Abbreviations: TSH, thyrotropin; MOM, multiples of the median; HR, hazard ratios. The specific number of knots of each model: A: 5; B:4; C:3; D:4. [file 13044_2024_192_MOESM3_ESM.pdf]

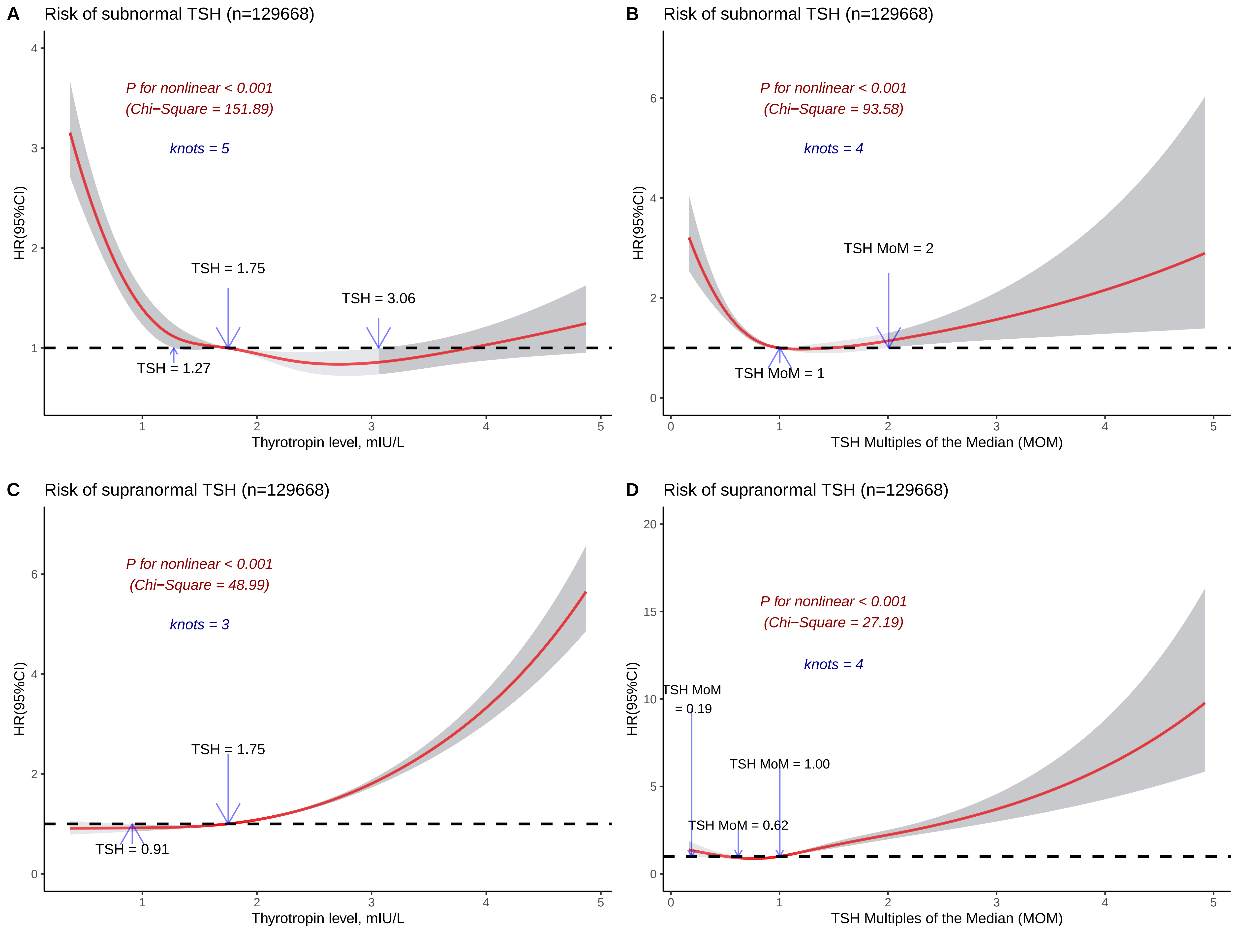
A
Risk of subnormal TSH (n=129668)
P for nonlinear < 0.001
(Chi−Square = 151.89)
knots = 5
TSH = 1.75
TSH = 3.06
TSH = 1.27
HR(95%CI)
Thyrotropin level, mIU/L
B
Risk of subnormal TSH (n=129668)
P for nonlinear < 0.001
(Chi−Square = 93.58)
knots = 4
TSH MoM = 2
TSH MoM = 1
HR(95%CI)
TSH Multiples of the Median (MOM)
C
Risk of supranormal TSH (n=129668)
P for nonlinear < 0.001
(Chi−Square = 48.99)
knots = 3
TSH = 1.75
TSH = 0.91
HR(95%CI)
Thyrotropin level, mIU/L
D
Risk of supranormal TSH (n=129668)
P for nonlinear < 0.001
(Chi−Square = 27.19)
knots = 4
TSH MoM
= 0.19
TSH MoM = 1.00
TSH MoM = 0.62
HR(95%CI)
TSH Multiples of the Median (MOM)
